# GENETIC PLEIOTROPY OF KIDNEY FUNCTION AND SOLUBLE RECEPTOR FOR AGE: THE LONG LIFE FAMILY STUDY

**DOI:** 10.1093/geroni/igad104.3368

**Published:** 2023-12-21

**Authors:** Mary Feitosa, Shiow Jin, Sandeep Acharya, Mary Wojczynski, Kaare Christensen, Joseph Zmuda, Michael Brent, Michael Province

**Affiliations:** Washington University School of Medicine, St. Louis, Missouri, United States; Washington University at St Louis, St. Louis, Missouri, United States; Washington University at St Louis, St. Louis, Missouri, United States; Washington University School of Medicine, St. Louis, Missouri, United States; University of Southern Denmark, Odense, Syddanmark, Denmark; University of Pittsburgh, Pittsburgh, Pennsylvania, United States; Washington University at St Louis, St. Louis, Missouri, United States; Washington University School of Medicine, St. Louis, Missouri, United States

## Abstract

Chronic kidney disease (CKD) patients have increased inflammation and oxidative stress that may escalate the production of advanced glycation end-products (AGEs), a biomarker implicated in aging. Circulate soluble receptor for AGE (sRAGE) is associated with CKD and inversely associated with the estimated glomerular filtration rate (eGFR). In the Long Life Family Study, eGFR measured by creatinine (eGFRcr) and cystatin-C (eGFRcys) correlated with sRAGE (r~ -0.27, p< 1x10-30). We investigated genetic pleiotropy conducting GWAS for eGFRcr, eGFRcys, and sRAGE and correlated meta-analysis (CMA) on GWAS p-values from whole-genome sequence variants in 4,182 subjects (age range: 24–110). Of the 59 loci identified (p< 5x10-8), 42 were novel eGFR discoveries. We performed TWAS and CMA-TWAS for eGFRcr, eGFRcys, and sRAGE on blood RNA-seq in 1,209 subjects and identified 17 genes with pleiotropic expressions for eGFRs and sRAGE (Bonferroni-p< 2.73x10-6). Several CMA-GWAS gene variants (e.g., SH3GLB1/SELENOF, CENPP) were eQTLs in glomeruli and tubule kidney (Human Kidney eQTL Atlas, HK-Atlas) and other tissues (GTEx-v8). The bioinformatics analyses identified (i)genes harboring eQTLs in glomeruli and tubule kidney (HK-Atlas), (ii)enhancer-promoter gene variants associated with kidney function-related phenotypes at genome-wide significance (GeneHancer/GeneCards/GWAS-catalog), and (iii)enhancer regions predicting gene expressions in kidney related-function (EPRI, enhancer-promoter-RNA maps). Some functional genes were found among bioinformatics (e.g., MTX1, TOPORS, SNTB1, LSP1, SNTB1, ATG2A, TP53INP2) and others were unique (e.g., TEC, CCN1, and ERGIC1, GeneHancer/GeneCards/GWAS-catalog). Genomic, transcriptomic, and bioinformatics results demonstrated evidence that some novel variants and genes have functional regulatory features in the kidney and are likely involved in biological and clinical aging.

